# PI3K inhibition enhances the anti-tumor effect of eribulin in triple negative breast cancer

**Published:** 2019-06-04

**Authors:** Sandeep Rajput, Zhanfang Guo, Shunqiang Li, Cynthia X. Ma

**Affiliations:** ^1^ Section of Medical Oncology, Division of Oncology, Department of Internal Medicine, Washington University School of Medicine, St. Louis, MO 63110, USA; ^2^ Siteman Cancer Center, Washington University School of Medicine, St. Louis, MO 63110, USA

**Keywords:** PI3K inhibitor, BKM120, eribulin, triple-negative breast cancer, patient-derived xenograft

## Abstract

Loss of the tumor suppressor phosphatase and tensin homolog (PTEN) is commonly observed in triple negative breast cancer (TNBC), leading to activation of the phosphoinositide 3-kinase (PI3K) signaling to promote tumor cell growth and chemotherapy resistance. In this study, we investigated whether adding a pan-PI3K inhibitor could improve the cytotoxic effect of eribulin, a non-taxane microtubule inhibitor, in TNBC patient-derived xenograft models (PDX) with loss of PTEN, and the underlying molecular mechanisms. Three TNBC-PDX models (WHIM6, WHIM12 and WHIM21), all with loss of PTEN expression, were tested for their response to BKM120 and eribulin, alone or in combination *in vivo*. In addition, the effect of drug treatment on cell proliferation and cell cycle progression were also performed *in vitro* using a panel of TNBC cell lines, including 2 derived from PDX models. The combination of eribulin and BKM120 led to additive or synergistic anti-tumor effect in 2 of the 3 PDX models, accompanied by an enhanced mitotic arrest and apoptosis in sensitive PDX models. In addition, the combination was synergistic in reducing mammosphere formation, and markers for epithelial-mesenchymal transition (EMT). In conclusion, PI3K inhibition induces synergistic anti-tumor effect when combined with eribulin, by enhancing mitotic arrest and apoptosis, as well as, reducing the cancer stem cell population. This study provides a preclinical rationale to investigate the therapeutic potential for the combination of PI3K inhibition and eribulin in the difficult to treat TNBC. Further studies are needed to identify the biomarkers of response for target patient selection.

## INTRODUCTION

Triple negative breast cancer (TNBC), defined by the lack of estrogen receptor (ER), progesterone receptor (PR) and HER2 gene amplification, represents approximately 15 to 20% of all breast cancer cases and is associated with the worst prognosis compared to other breast cancer subtypes [1-6]. The lack of molecularly targeted therapy and the frequent occurrence of chemotherapy resistance render TNBC a significant clinical challenge.

Eribulin mesylate (Halaven®, Eisai Inc) is a non-taxane microtubule dynamic inhibitor [7] that is Food and Drug Administration (FDA) approved for patients with metastatic breast cancer who have received at least two prior chemotherapeutic regimens in the metastatic setting and an anthracycline as well as a taxane in either adjuvant or metastatic setting. In the phase III EMBRACE study, eribulin was associated with superior overall survival (OS) compared with physician's choice chemotherapy in this patient population [8]. The efficacy of eribulin in TNBC was demonstrated in the pooled analysis of 2 phase 3 studies (EMBRACE/Study 305), which revealed 4.7 months improvement in median survival with eribulin compared to control chemotherapy (median OS: 12.9 vs 8.2 months; HR 0.74; *P* = 0.006) [9]. In addition to the induction of an irreversible mitotic block, eribulin has been shown to impact tumor vascular remodeling [10] and inhibition of epithelial-to-mesenchymal transition and metastasis in experimental models [11] which has been implicated in therapeutic resistance to cancer drugs including growth factor receptor and PI3K inhibitors [12].

The phosphoinositide 3-kinase (PI3K) pathway plays key regulatory roles in many cellular processes, including cell survival, proliferation, differentiation and angiogenesis [13, 14]. Hyper activation of the PI3K/AKT pathway has been associated with TNBC [15, 16]. A significantly higher level of Akt phosphorylation has been observed in TNBC patient specimens compared with non–TNBC cases [15, 17]. Loss of PTEN or INPP4B has been the most frequently implicated culprit for such activation in TNBC [16, 18-21]. The high frequency of PI3K pathway activation in TNBC renders it an attractive therapeutic target. In addition, PI3K pathway activation has also been associated with chemotherapy resistance [22] and inhibition of PI3K pathway activity could synergize the cytotoxicity of a variety of chemotherapy agents [23-25]. In a cell-based, high-throughput screening in a panel of twenty-five human cancer cell lines representing a variety of tumor types, the PI3K inhibitor BKM120 was identified to exert synergistic killing with eribulin in both eribulin sensitive and resistant cancer cell lines, 3 of which being TNBC [26]. The objectives of this study is to assess the combinatory effect of eribulin and BKM120 in TNBC cell lines and patient-derived xenograft (PDX) models and to further elucidate the underlying molecular mechanisms.

## RESULTS

### Synergistic anti-tumor effect of eribulin and BKM120 through enhanced target inhibition in a panel of TNBC cell lines

**Figure 1:**
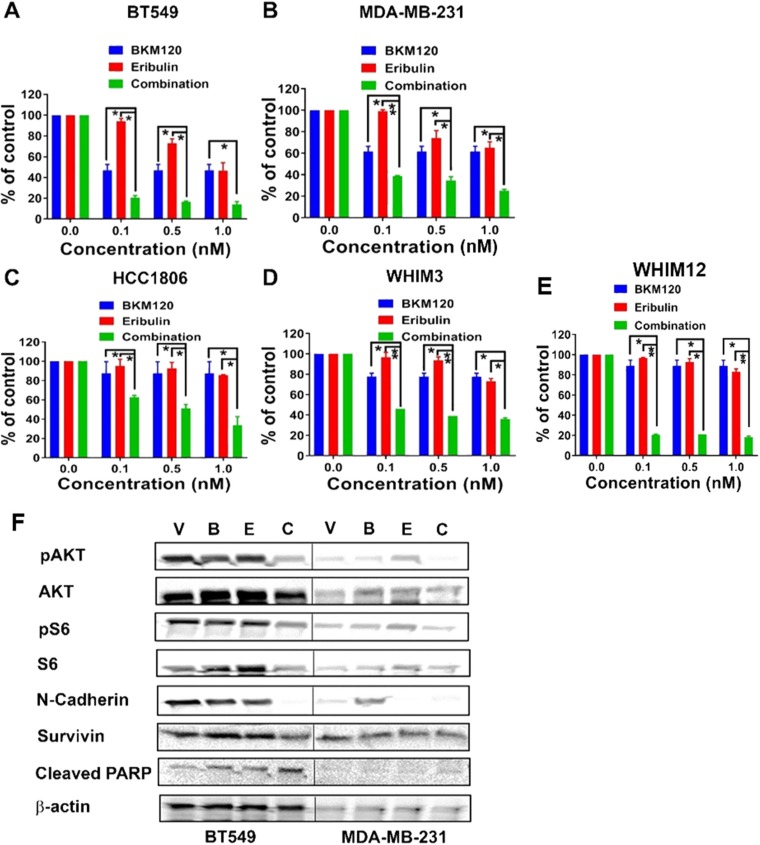
Eribulin in combination with BKM120 induces synergistic anti-tumor effect and target inhibition in TNBC cells *in vitro*. Percentages of cell survival compared to vehicle control following 6 days of treatment with eribulin at indicated concentrations, either alone or in combination with BKM120 (500 nM) were plotted for **(A)** BT549, **(B)** MDA-MB-231, **(C)** HCC1806, **(D)** WHIM3, and **(E)** WHIM12. ^*^ indicates p<0.05 and ^**^ indicates p<0.01. The combination of BKM120 and eribulin was significantly more effective in reducing cell survival than each agent alone in these cell lines. Panel **F** shows the Western blot analysis for PI3K pathway signaling, EMT, and apoptosis markers on cell lysates from BT549 and MDA-MB-231 following treatment with eribulin and BKM120, alone or in combination for 48 hours. Abbreviations: V, Vehicle; B, BKM120; E, Eribulin; C, Combination. Compared to single agents, the combination of BKM120 and eribulin was most effective in reducing the levels of pAKT and pS6 (markers of PI3K pathway activity), N-Cadherin (EMT marker) and Survivin (an anti-apoptotic protein) and in the induction of apoptosis (Cleaved PARP).

**Figure 2:**
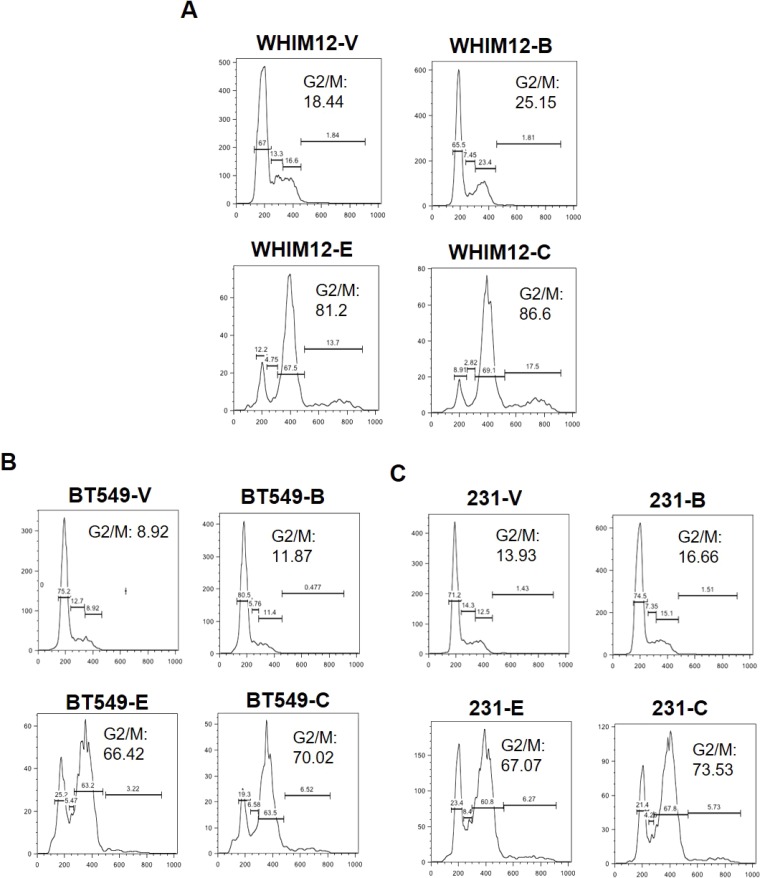
Eribulin in combination with BKM120 induces G2/M cell cycle arrest more effectively than either agent alone in TNBC. Cell Cycle analysis was performed for **(A)** WHIM12-PDX derived cell line, **(B)** BT549 and **(C)** MDA-MB-231 TNBC cell lines after treatment with vehicle, or BKM120 and eribulin, alone and in combination. Abbreviations: V, Vehicle; B, BKM120; E, Eribulin; C, Combination. The combination of eribulin and BKM120 was most effective in inducing G2/M arrest.

To assess the anti-tumor effect of BKM120 and eribulin, we tested a panel of TNBC cell lines (BT549, HCC1806, and MDA-MB-231) as well as two PDX derived cell lines (WHIM3 and WHIM12), for their response to eribulin (0.1, 0.5 and 1nM) alone or in combination with BKM120 (500nM) *in vitro*. Synergistic anti-tumor effect, with combination index (CI) less than 1, between eribulin and BKM120 was observed in all the tested TNBC cell lines (Figure 1A-1E). Western Blot demonstrated that, compared to individual agents, the combination of eribulin and BKM120 was most effective in inhibiting PI3K pathway signaling, as indicated by reduced pAKT and pS6 in BT549 and MDA-MB-231 cells (Figure 1F). Additionally, the combination reduced the level of the Epithelial to Mesenchymal Transition (EMT) marker N-Cadherin more effectively than eribulin alone, reduced the levels of the anti-apoptotic protein Survivin, and enhanced apoptosis as assessed by cleaved PARP (Figure 1F). Since eribulin induces apoptosis by inhibiting mitotic progression, we performed cell cycle analysis with flow cytometry, which demonstrated that the combination of eribulin and BKM120 was most effective in inducing G2/M arrest (Figure 2).

### Anti-tumor activity of eribulin in combination with BKM120 in TNBC PDX models *in vivo*

**Figure 3:**
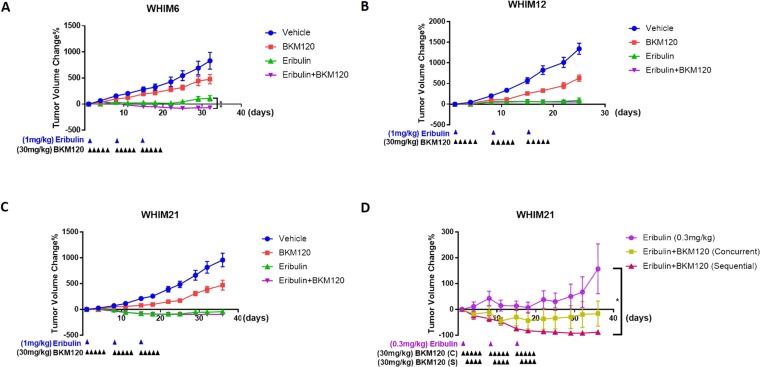
Eribulin in combination with BKM120 inhibits tumor growth in TNBC PDXs. Tumor volume changes with time compared to baseline following treatment with either vehicle, eribulin (1 mg/kg, IP, day 1 each week x 3), BKM120 (30 mg/kg, PO, days 1-5 each week x3) or the combination of eribulin and BKM120 in **(A)** WHIM6, **(B)** WHIM12 and **(C)** WHIM21 TNBC PDX models *in vivo* (n=6). **(D)** Tumor volume changes with time compared to baseline following treatment with either eribulin (0.3 mg/kg, IP, day 1 each week x 3), or the combination of eribulin and BKM120 administered concurrently (eribulin 0.3 mg/kg, IP, day 1 and BKM120 30 mg/kg, PO, days 1-5 each week) or sequentially (eribulin 0.3 mg/kg, IP, day 1 followed by BKM120 30 mg/kg, PO, on days 2-5 each week) in WHIM21. ^*^ indicates p<0.05 and ^****^ indicates p<0.0001.

To validate the *in vitro* observation we evaluated the anti-tumor and biomarker effect for eribulin and BKM120 in TNBC PDX models that we have previously characterized [27]. We first performed a screening experiments using 1-3 mice per model for the combination of eribulin and BKM120 in 6 TNBC PDX models, including WHIM2, WHIM4, WHIM6, WHIM12, WHIM21, and WHIM30 (Supplementary Figure 1). As shown in Figure 3A, tumor volume reduction was observed in 5 of the 6 models, including WHIM2 (average -21% on day 11), WHIM4 (average -25% on day 15), WHIM6 (average -18% on day 11), WHIM21 (average -92% on day 18) and WHIM30 (average -66% on day 22) compared to baseline at the best response. To discern the effect of single agent versus combination, we treated 3 representative models including, WHIM6 (Basal-like, WT TP53), WHIM12 (Claudin-low, TP53 p.R248Q, PIK3CA pV105_E109delinsT) and WHIM21 (Basal-like, TP53 p.P151H), all with loss of PTEN expression and relatively high PI3K pathway signaling [27] to either vehicle, eribulin, BKM120, or the combination of eribulin and BKM120. Combination therapy led to added or synergistic anti-tumor effect in WHIM6 (Figure 3A). However, no obvious added benefit was observed with the combination compared to eribulin alone in WHIM12 and WHIM21 (Figure 3B and 3C). Since eribulin alone at 1 mg/kg weekly dosing potently inhibited xenograft tumor growth, which could have prohibited further tumor growth inhibition with the addition of BKM120, we reduced the dose of eribulin to 0.3 mg/kg weekly in WHIM21 to compare its anti-tumor effect with or without BKM120. Indeed, the addition of BKM120 to the lower dose of eribulin, either given concurrently (eribulin on day 1 and BKM120 on days 1-5, each week) or sequentially (eribulin on day 1 and BKM120 on days 2-5, each week) led to more effective tumor growth inhibition compared to eribulin alone (Figure 3D).

### The combination of eribulin and BKM120 enhanced mitotic arrest and apoptotic induction in TNBC PDX models *in vivo*

**Figure 4:**
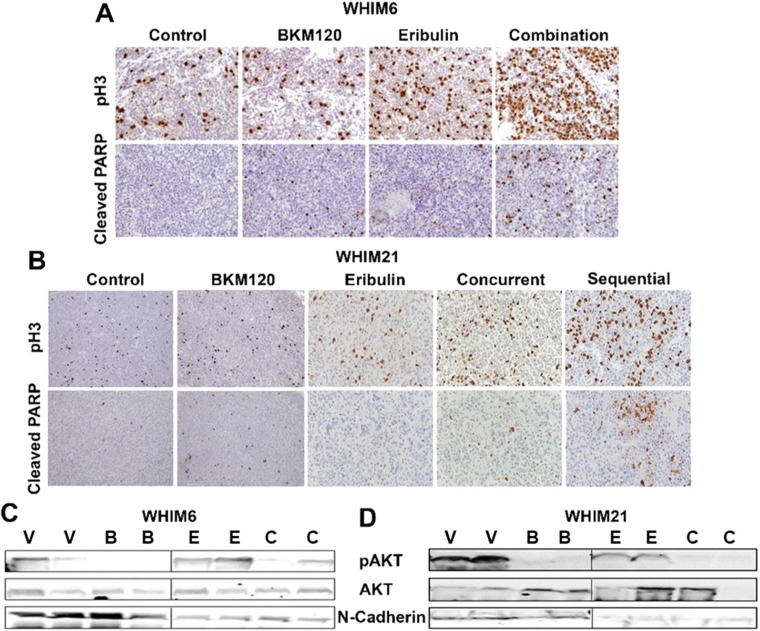
Eribulin in combination with BKM120 induces more apoptosis, mitotic cell cycle arrest and target inhibition in TNBC PDXs. Representative pictures for IHC analysis of Cleaved-PARP and phospho-histone3 (pH3) following 3 days of therapy with either vehicle or eribulin and BKM120, alone or in combination are shown for **(A)** WHIM6 and **(B)** WHIM21 PDX tumors. Combination therapy was associated with enhanced mitotic arrest (pH3) and apoptosis (Cleaved PARP). Western blot analysis for pAKT, AKT and N-Cadherin was performed on post treatment tumor lysates for **(C)** WHIM6 and **(D)** WHIM21. Abbreviations: V, Vehicle; B, BKM120; E, Eribulin; C, Combination. Treatment with single agent BKM120 and eribulin reduced the levels of pAKT and N-Cadherin, respectively. The combination of BKM120 and eribulin reduced the levels of both pAKT and N-Cadherin.

To assess the molecular mechanisms for the enhanced anti-tumor effect from the combination of BKM120 and eribulin in TNBC *in vivo*, PDX xenografts were harvested on day 3 following treatment with either vehicle or eribulin (day 1) and BKM120 (days 1-3) alone or in combination and subjected to biomarker analysis. Consistent with the *in vitro* observation for the enhanced G2/M phase arrest and apoptosis induction (Figure 1E and Figure 2), combination therapy led to more effective mitotic arrest, as shown by the increased proportion of cells with pHistone H3 staining, and increased apoptosis (increased cleaved PARP) *in vivo* (Figure 4A and 4B). As predicted, treatment with BKM120 reduced PI3K pathway signaling indicated by the levels of pAKT and treatment with eribulin inhibited EMT marker N-cadherin (Figure 4C and 4D) as observed *in vitro* (Figure 1E).

### Eribulin and BKM120 synergistically inhibits mammosphere formation and stem cell population

**Figure 5:**
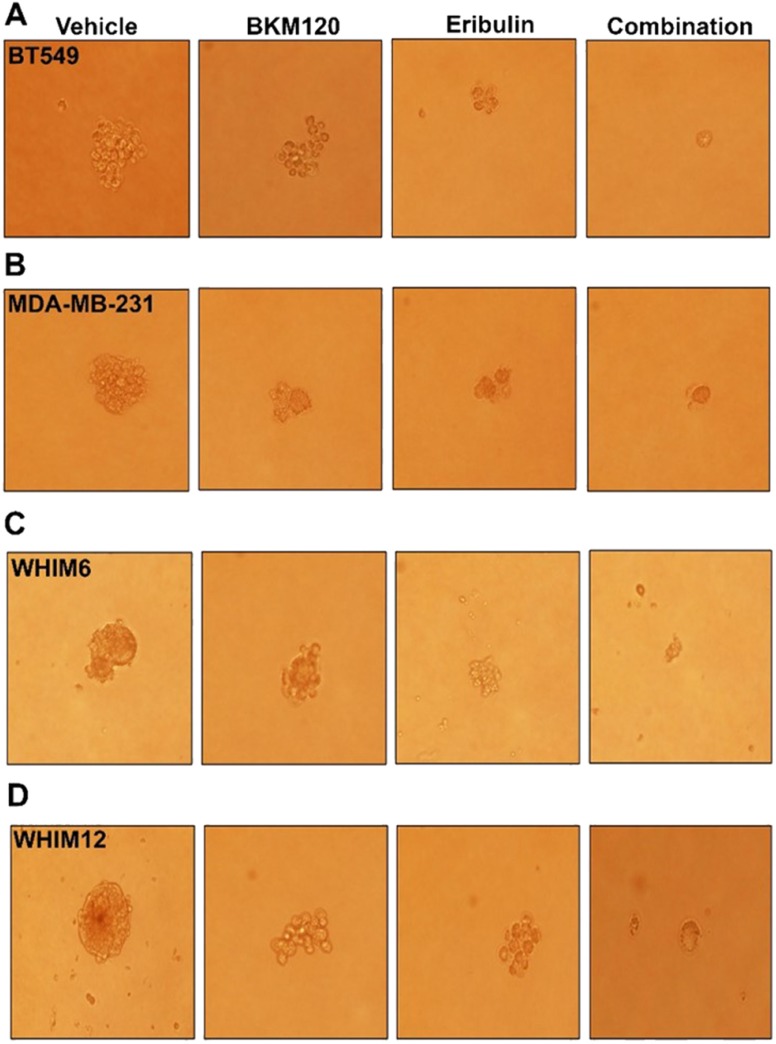
Eribulin alone or in combination with BKM120 inhibited mammosphere formation in TNBC cell lines *in vitro*. Mammosphere formation was assessed following 6 days treatment with either vehicle, eribulin, BKM120, or the combination of eribulin and BKM120 for **(A)** BT549, **(B)** MDA-MB-231, **(C)** ex vivo cultured WHIM6 and **(D)** WHIM12 PDX derived cell line. Representative photographs were shown. The combination of eribulin and BKM120 was most in inhibiting mammosphere formation.

**Figure 6:**
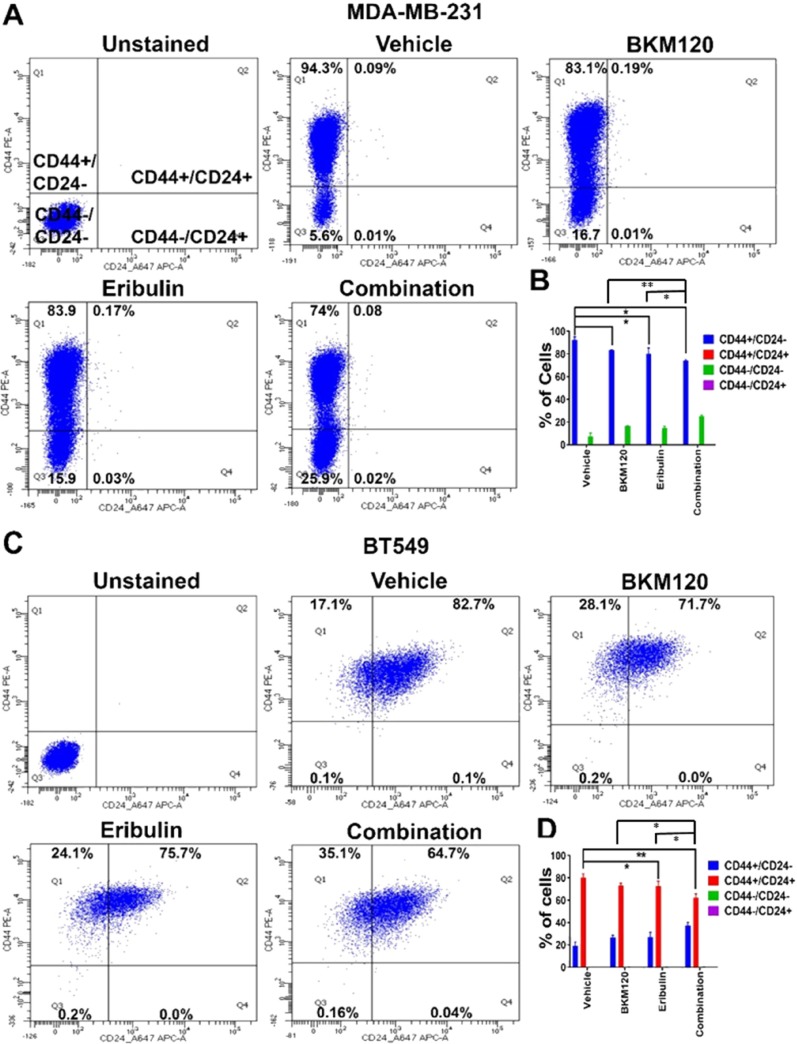
Eribulin in combination with BKM120 inhibited stem cell population in TNBC lines *in vitro*. Representative FACS analysis of stem cell population in **(A)** MDA-MB-231 and **(C)** BT549 cells after eribulin and BKM120 single and combination treatment for 48 hours. Gates were adjusted using unstained and isotype secondary controls. **(B)** and **(D)** shows the quantification of cellular subsets by CD44 and CD24 staining for MDA-MB-231 and BT549, respectively, from three independent experiments. ^*^ indicates p<0.05 and ^**^ indicates p<0.01.

The effect of eribulin and BKM120 on N-cadherin in TNBC cell lines suggest that the combination is synergistic in reducing EMT. As EMT is associated with cancer cell stemness, we hypothesize that the combination therapy is synergistic in reducing the cancer stem cell population. We, therefore, examined the effect of eribulin and BKM120, alone or in combination, on mammosphere formation in low attachment plates as well as on CD44^+^/CD24^-neg/low^ sub-population, the putative stem cell population, in TNBC cell lines. As demonstrated in Figure 5, the combination of eribulin and BKM120 inhibited mammosphere formation synergistically in BT549, and MDA-MB-231, as well as, in the ex-vivo cultured WHIM6 and WHIM12-PDX cells. In addition, eribulin in combination with BKM120 was more effective than each agent alone in reducing the percentage of the CD44^+^/CD24^neg^ sub-population in MDA-MB-231 cells (Figure 6A, 6B). Interestingly, in BT549 cells, treatment with eribulin and BKM120 reduced the percentage of CD44^+^/CD24-population, with combination therapy being the more effective (Figure 6C, 6D). Co-expression of CD44 and CD24 is frequently observed in basal/epithelial breast cancer cells [28] and CD44^+^/CD24^+^ cells have shown to be more invasive and tumorigenic than CD44^+^/CD24^neg^ cells and possess stemness characteristics of self-renewal and differentiation in multiple cancer types [29, 30]. This is in contrast to paclitaxel, another microtubule inhibitor, which did not inhibit these subsets of cell population, either administered alone or in combination with BKM120 (Supplementary Figure 2).

## DISCUSSION

Resistance to chemotherapy is associated with poor clinical outcomes in patients with TNBC, for which targeted therapies are lacking [1, 31-32]. In this study, we demonstrated that PI3K inhibition in combination with an anti-mitotic chemotherapy agent eribulin led to enhanced target inhibition, reduction of cancer stem cell population, and synergistic cytotoxic effect in TNBC cell lines and PDX models, providing a preclinical rationale for further clinical investigation.

PI3K pathway signaling plays key regulatory roles in many cellular processes, including cell survival, proliferation, differentiation and angiogenesis [13, 14]. Aberrant activation of the PI3K pathway signaling is frequently observed in TNBC [15, 21]. Compared to other subtypes, TNBC is associated with significantly higher levels of AKT phosphorylation, as well as, PI3K signaling activity assessed by either gene expression signature or RPPA phosphoproteomic signature [16, 17, 33]. Common PI3K pathway gene abnormalities identified in the TCGA TNBC data set included PIK3CA mutation (7%), as well as, loss/mutations of PTEN (35%) and loss of INPP4B (16%), which are the major causes of pathway signaling activation [33]. Consistent with the importance of PI3K pathway in TNBC tumorigenesis, PTEN inactivation leads to “basal-like” breast cancer in animal models [34, 35].

PI3K pathway signaling activation has been identified to be a key resistance mechanism to chemotherapy as demonstrated in a recent study which investigated genomic alterations and gene expression profiles associated with chemotherapy resistance in patients with TNBC [36]. In addition, inhibition of PI3K pathway components has been shown to enhance the sensitivity to various chemotherapy agents in preclinical models [26, 37-42]. For example, significant synergisms in terms of reducing cell proliferation and induction of apoptosis were observed with the combination of ipatasertib, an AKT inhibitor, or taselisib, a PI3K inhibitor, and anti-microtubule agents including paclitaxel, vinorelbine, and eribulin in PIK3CA mutant breast cancer cell lines *in vitro* [40]. Although the first-generation mTOR or PI3K inhibitors have generated mixed results when combined with paclitaxel in clinical trials [43], more encouraging data has been observed with direct AKT inhibitors in the neoadjuvant [44], as well as, metastatic settings for the treatment of TNBC [45]. In this paper, we show that BKM120 enhances the cytotoxic effect of the non-taxane microtubule inhibitor eribulin in TNBC PDX models with loss of PTEN, accompanied by enhanced mitotic arrest and apoptosis induction. Our data provides *in vivo* validation for the synergistic effect of this combination observed in a cell-based assay [26] and is in line with a prior study presented in an abstract form in PIK3CA mutant breast cancer xenograft models [42]. These results are in support of ongoing and future clinical trial investigations of eribulin with PI3K pathway inhibitors such as NCT02723877 and NCT02616848 in this difficult to treat patient population.

We were particularly interested in eribulin as chemotherapy agent because of its unique mechanisms of action and potential effect on EMT. There is increasing evidence in the literature indicating that CSCs play a crucial role in therapy resistance [46-48] and that activation of the PI3K/AKT pathway is indispensable for maintaining the stemness and chemoresistance of breast CSCs [49, 50]. Moreover, previous studies have also shown that PI3K inhibition sensitizes CSCs to chemotherapy and molecular targeted therapy in several cancers including leukemia, hepatocellular carcinoma and breast cancer [51-53]. In this study, we showed that BKM120 and eribulin each reduced mammosphere formation and CSC population as a single agent *in vitro*, but the combination was more effective. We demonstrated that the combination of eribulin and BKM120 was most effective in inhibiting downstream signaling of PI3K and EMT markers, accompanied by enhanced mitotic arrest and apoptosis induction.

The limitations of this study includes the small number of PDX models tested and the lack of predictive marker biomarkers for the combination of eribulin and BKM120. A larger cohort of PDX models are needed for biomarker development. In addition, BKM120 is no longer being developed clinically due to its associated side effects. However, this is a proof of concept study demonstrating the potential of eribulin and PI3K inhibitor combinations. Future studies will focus on other clinical PI3K inhibitors.

## MATERIALS AND METHODS

### Chemicals

BKM120 (Catalog no. CT-BKM120) was purchased from Chemietek. Eribulin was provided by Eisai. Paclitaxel (Catalog no. S1150) was purchased from Selleckchem. All drugs were prepared in stock solution of 10 mM in dimethyl sulfoxide (DMSO; Sigma) for *in vitro* experiments.

### *In vitro* cytotoxic assay

For cytotoxic assay, BT549, MDA-MB-231, HCC1806, WHIM3, and WHIM12 were seeded at a density of 3,000 cells per well in 96-well plates in RPMI-1640 medium supplemented with 10% FBS, 1% glutamine, and 1% penicillin-streptomycin for 24 hours. Cells were treated with vehicle, or varying doses of eribulin (0-1 nM) either alone or in combination with BKM120 (500 nM) for 6 days followed by Alamar Blue Assay. Each experiment was repeated twice in triplicate. Synergistic or additive activity between eribulin and BKM120 was determined by calculating combination index values using Compu Syn software.

### Cell lines

BT549, MDA-MB-231, and HCC1806 were purchased from ATCC. WHIM3 (also named WU-BC3) and WHIM12 were established from PDX models and were described previously [27, 54].

### Western blotting

Cell lines treated with eribulin or BKM120 were seeded in a 6-well plate at a density of 0.5 × 10^6^, adhered overnight and treated with DMSO (0.1%), eribulin at 5nM and BKM120 at 500 nM concentration for 48 hours. Cells were then harvested and lysed with 100 μL buffer containing 50 mmol/L Tris-HCl, pH 7.5, 150 mmol/L NaCl, 2 mmol/L EDTA, 1% Triton, 1 mmol/L phenylmethylsulfonylfluoride, and Protease Inhibitor Cocktail (Sigma) for 20 min on ice. Lysates from cell lines or PDX xenografts were cleared at 10,000 rpm for 15 min, boiled, separated on 12% SDS gels, and transferred to a nitrocellulose membrane followed by overnight incubation with primary antibodies against pAKT^473^, AKT, pS^6240/244^, S6, N-Cadherin, cleaved PARP, survivin (BRIC5) and β-actin. Protein bands were visualized after 1 hour incubation with HRP-conjugated secondary antibodies and development with ECL (GE Healthcare).

Primary antibodies against phospho-AKT^S473^ (Cat. no. 4060), AKT (Cat. no. 4685), phospho- S6^Ser240/244^ (Cat. no. 2215), S6 (Cat. no. 2217), Cleaved PARP (Asp214) (Cat. no. 9541), and β-actin (Cat. no. 4970) were purchased from Cell Signaling Technology. Other primary antibodies included antibodies against N-Cadherin (Cat. no. ab18203; Abcam), survivin (Cat. no. sc-17779; Santa Cruz), and p21 (Cat. no. sc-6246; Santa Cruz). Secondary horseradish peroxidase (HRP)-conjugated anti-rabbit (Cat.7074) and anti-mouse antibodies (Cat. no. 7076) for Western Blot were purchased from Cell Signaling Technology.

### Flow cytometry cell cycle analysis

BT549, MDA-MB-231 and WHIM12 cells were seeded in 6-well plate at a density of 500,000 cells/well in 2ml RPMI medium. After 24 hours, cells were treated with vehicle, or BKM120 (500nM) and eribulin (5nM), either alone or in combination for 48 hours. Cells were then harvested and analyzed for cell cycle analysis using Invitrogen Propidium Iodide staining buffer. Data from three independent experiments were used for the quantification of cellular subsets in Figure 6B, Figure 6D, Supplementary Figure 2B, and Supplementary Figure 2D.

### Immunohistochemistry

IHC for pHistone H3 and cleaved PARP were conducted on 5 μm tissue sections from paraffin-embedded tumor as described previously using the EnVision + Single Reagents HRP-Rabbit (Dako) and REAL substrate buffer (REAL DAB + chromogen, Dako) [54]. The primary antibodies and dilutions are as follows: pHistone H3 (Ser 10) antibody (1:200; Cat. no. 06-570; Millipore), and cleaved PARP antibody (1:200, Cat. no. 9541; Cell Signaling).

### Analysis of CD44+ /CD24– cell subpopulation

Cells were seeded in a 6-well plate at a density of 0.5 × 10^6^, adhered overnight, followed by treatment with DMSO (0.1 %), eribulin at 5nM and BKM120 at 500 nM concentration for 48 hours. After 48 hours, cells were harvested for CD44/C24 expression analysis by FACS. To evaluate CD24 and CD44 expression, cells were harvested, washed with FCS buffer once. Then, 100,000 cells again resuspended in 1ml FCS buffer and antibodies against CD44-PE (10ul) (Cat. no. 555479; BD Pharmingen) and CD24 Alexa-Flour 647- (2.5ul) (Cat. no. 311109; BioLegend) were added for 30 minutes at room temperature in the dark. After 30 minutes, cells were washed with PBS two times and resuspended in 1ml FCS buffer and analyzed by FACS. Then cells were analyzed on a BD FACS Calibur flow cytometer. These data were analyzed by Flo-Jo and at least 10,000 events per sample were collected. Appropriate controls were included in the experiments. Unstained samples, Isotype-Secondary control, CD44 and CD24 alone staining.

### Mammosphere culture

Sphere formation was performed in ultralow attachment 6-well plates (Corning) with DMEM/F12 stem cell medium. (Thermo scientific). BT549, MDA-MB-231, WHIM12 and WHIM6-PDX derived cells were seeded at the density around 1000 cells/well in ultralow attachment 6-well plates and cultured at 37°C in 5% CO2. After 24 hours, cells were treated with vehicle, BK1M20 (500nM) and Eribulin (5nM) for 6 days. Colony formation was assessed and photomicrograph after 6 days of treatment.

### *In vivo* xenografts-drug therapy and assessment

PDX models were passaged on each side of the fourth mammary fat pad in female NU/J homozygous mice (Charles River Cat. No. 088) to propagate xenografts for tumor growth and biomarker response with drug therapy. For the initial screening experiments, 6 PDX models with each passaged in 1-3 mice were allowed to grow to approximately 1 cm in diameter, then treated with eribulin (0.75 mg/kg, IP days 1, 3 and 8) and BKM120 (30 mg/kg, PO, days 1-5 then days 7-8). For tumor growth experiments shown in Figure 3A-3C, xenografts were allowed to grow to approximately 0.5 cm in the maximum diameter. Mice were then divided into 4 treatment groups (*n* = 6 mice in each group): Vehicle (vehicle diluents were 0.9% sodium chloride saline for eribulin and 0.5% (w/v) methyl cellulose 0.5% (v/v) Tween 80 in water for BKM120), eribulin alone (1 mg/kg, IP on day 1 each week), BKM120 alone (30mg/kg, oral gavage, days 1-5 each week) or the combination of eribulin and BKM120 (at the same dose and schedule as in single agent therapy) for a total of 3 weeks. For WHIM21 tumor response shown in Figure 3D, tumor bearing mice (n=6 per group) were treated with either eribulin (0.3mg/kg) or the combination of eribulin and BKM120 concurrently (eribulin 0.3 mg/kg, IP, day 1 and BKM120 30 mg/kg, PO, days 1-5 each week) or sequentially (eribulin 0.3 mg/kg, IP, day 1 followed by BKM120 30 mg/kg, PO, on days 2-5 each week) for 3 weeks. Two dimensional measurements (length and width) using Traceable Digital Calipers were performed 2-3 times each week. The following formula was used to calculate tumor volume: tumor volume (cm^3^) = (length × width^2^) × 0.5. For biomarker studies tumor bearing mice were treated with either Vehicle (*n* = 2), eribulin (n=2; 1 mg/kg/day,IP day1), BKM120 (n=2, 30 mg/kg, days 1, 2, 3), or the combination of eribulin and BKM120 (the same dosing and schedule as single agent therapy). Tumors were harvested 2 hours after day 3 of therapy. Each xenograft tumor was cut into 2 pieces with one piece flash frozen for tumor lysate, and the second piece fixed in 10% neutral buffered formalin and embedded in paraffin blocks. All animal studies were carried out using the appropriate NIH animal care, and the animal studies protocol was approved by the Animal Studies Committee of Washington University.

### Statistical analysis

Statistical analyses were conducted using Graphpad Prism software. Results are expressed as mean ± SEM. Statistical significance was determined by Student paired *t* test for *in vitro* data respectively. Tumor volume data were compared using two-way ANOVA. *P* ≤ 0.05 was considered significant.

## SUPPLEMENTARY MATERIALS FIGURES



## Abbreviations

PI3K: Phosphatidylinositol-3-Kinase
PTEN: Phosphatase and tensin homolog
TNBC: triple negative breast cancer
PDX: patient derived xenograft
WHIM: Washington University Human-in-Mouse
EMT: epithelial-mesenchymal transition
ER: estrogen receptor
PR: progesterone receptor
HER2: human epidermal growth factor receptor 2
FDA: Food and Drug Administration
RPPA: reverse phase protein array
NU/J: homozygous nude
IP: intraperitoneal injection
PO: per os (meaning by mouth)
NIH: National Institutes of Health
SEM: standard error of the mean

## References

[1] Foulkes WD, Smith IE, Reis-Filho JS. Triple-negative breast cancer. N Engl J Med. 2010; 363:1938–48. 10.1056/NEJMra1001389 21067385

[2] Hudis CA, Gianni L. Triple-negative breast cancer: an unmet medical need. Oncologist. 2011; 16:1–11. 10.1634/theoncologist.2011-S1-01 21278435

[3] Lehmann BD, Bauer JA, Chen X, Sanders ME, Chakravarthy AB, Shyr Y, Pietenpol JA. Identification of human triple-negative breast cancer subtypes and preclinical models for selection of targeted therapies. J Clin Invest. 2011; 121:2750–67. 10.1172/JCI45014 21633166 PMC3127435

[4] Lehmann BD, Jovanović B, Chen X, Estrada MV, Johnson KN, Shyr Y, Moses HL, Sanders ME, Pietenpol JA. Refinement of Triple-Negative Breast Cancer Molecular Subtypes: Implications for Neoadjuvant Chemotherapy Selection. PLoS One. 2016; 11:e0157368. 10.1371/journal.pone.0157368 27310713 PMC4911051

[5] Shah SP, Roth A, Goya R, Oloumi A, Ha G, Zhao Y, Turashvili G, Ding J, Tse K, Haffari G, Bashashati A, Prentice LM, Khattra J, et al. The clonal and mutational evolution spectrum of primary triple-negative breast cancers. Nature. 2012; 486:395–99. 10.1038/nature10933 22495314 PMC3863681

[6] Carey LA, Dees EC, Sawyer L, Gatti L, Moore DT, Collichio F, Ollila DW, Sartor CI, Graham ML, Perou CM. The triple negative paradox: primary tumor chemosensitivity of breast cancer subtypes. Clin Cancer Res. 2007; 13:2329-34. 10.1158/1078-0432.CCR-06-1109 17438091

[7] Smith JA, Wilson L, Azarenko O, Zhu X, Lewis BM, Littlefield BA, Jordan MA. Eribulin binds at microtubule ends to a single site on tubulin to suppress dynamic instability. Biochemistry. 2010; 49:1331–37. 10.1021/bi901810u 20030375 PMC2846717

[8] Cortes J, O’Shaughnessy J, Loesch D, Blum JL, Vahdat LT, Petrakova K, Chollet P, Manikas A, Diéras V, Delozier T, Vladimirov V, Cardoso F, Koh H, et al, and EMBRACE (Eisai Metastatic Breast Cancer Study Assessing Physician’s Choice Versus E7389) investigators. Eribulin monotherapy versus treatment of physician’s choice in patients with metastatic breast cancer (EMBRACE): a phase 3 open-label randomised study. Lancet. 2011; 377:914–23. 10.1016/S0140-6736(11)60070-6 21376385

[9] Twelves C, Cortes J, Vahdat L, Olivo M, He Y, Kaufman PA, Awada A. Efficacy of eribulin in women with metastatic breast cancer: a pooled analysis of two phase 3 studies. Breast Cancer Res Treat. 2014; 148:553–61. 10.1007/s10549-014-3144-y 25381136 PMC4243003

[10] Funahashi Y, Okamoto K, Adachi Y, Semba T, Uesugi M, Ozawa Y, Tohyama O, Uehara T, Kimura T, Watanabe H, Asano M, Kawano S, Tizon X, et al. Eribulin mesylate reduces tumor microenvironment abnormality by vascular remodeling in preclinical human breast cancer models. Cancer Sci. 2014; 105:1334–42. 10.1111/cas.12488 25060424 PMC4462349

[11] Yoshida T, Ozawa Y, Kimura T, Sato Y, Kuznetsov G, Xu S, Uesugi M, Agoulnik S, Taylor N, Funahashi Y, Matsui J. Eribulin mesilate suppresses experimental metastasis of breast cancer cells by reversing phenotype from epithelial-mesenchymal transition (EMT) to mesenchymal-epithelial transition (MET) states. Br J Cancer. 2014; 110:1497–505. 10.1038/bjc.2014.80 24569463 PMC3960630

[12] Byers LA, Diao L, Wang J, Saintigny P, Girard L, Peyton M, Shen L, Fan Y, Giri U, Tumula PK, Nilsson MB, Gudikote J, Tran H, et al. An epithelial-mesenchymal transition gene signature predicts resistance to EGFR and PI3K inhibitors and identifies Axl as a therapeutic target for overcoming EGFR inhibitor resistance. Clin Cancer Res. 2013; 19:279–90. 10.1158/1078-0432.CCR-12-1558 23091115 PMC3567921

[13] Vivanco I, Sawyers CL. The phosphatidylinositol 3-Kinase AKT pathway in human cancer. Nat Rev Cancer. 2002; 2:489–501. 10.1038/nrc839 12094235

[14] Engelman JA. Targeting PI3K signalling in cancer: opportunities, challenges and limitations. Nat Rev Cancer. 2009; 9:550–62. 10.1038/nrc2664 19629070

[15] Marty B, Maire V, Gravier E, Rigaill G, Vincent-Salomon A, Kappler M, Lebigot I, Djelti F, Tourdès A, Gestraud P, Hupé P, Barillot E, Cruzalegui F, et al. Frequent PTEN genomic alterations and activated phosphatidylinositol 3-kinase pathway in basal-like breast cancer cells. Breast Cancer Res. 2008; 10:R101. 10.1186/bcr2204 19055754 PMC2656897

[16] López-Knowles E, O’Toole SA, McNeil CM, Millar EK, Qiu MR, Crea P, Daly RJ, Musgrove EA, Sutherland RL. PI3K pathway activation in breast cancer is associated with the basal-like phenotype and cancer-specific mortality. Int J Cancer. 2010; 126:1121–31. 10.1002/ijc.24831 19685490

[17] Umemura S, Yoshida S, Ohta Y, Naito K, Osamura RY, Tokuda Y. Increased phosphorylation of Akt in triple-negative breast cancers. Cancer Sci. 2007; 98:1889–92. 10.1111/j.1349-7006.2007.00622.x 17892507 PMC11158483

[18] Bose S, Chandran S, Mirocha JM, Bose N. The Akt pathway in human breast cancer: a tissue-array-based analysis. Mod Pathol. 2006; 19:238–45. 10.1038/modpathol.3800525 16341149

[19] Leary A, Dowsett M. Combination therapy with aromatase inhibitors: the next era of breast cancer treatment? Br J Cancer. 2006; 95:661–66. 10.1038/sj.bjc.6603316 16926831 PMC2360507

[20] Saal LH, Holm K, Maurer M, Memeo L, Su T, Wang X, Yu JS, Malmström PO, Mansukhani M, Enoksson J, Hibshoosh H, Borg A, Parsons R. PIK3CA mutations correlate with hormone receptors, node metastasis, and ERBB2, and are mutually exclusive with PTEN loss in human breast carcinoma. Cancer Res. 2005; 65:2554–59. 10.1158/0008-5472-CAN-04-3913 15805248

[21] Fedele CG, Ooms LM, Ho M, Vieusseux J, O’Toole SA, Millar EK, Lopez-Knowles E, Sriratana A, Gurung R, Baglietto L, Giles GG, Bailey CG, Rasko JE, et al. Inositol polyphosphate 4-phosphatase II regulates PI3K/Akt signaling and is lost in human basal-like breast cancers. Proc Natl Acad Sci USA. 2010; 107:22231–36. 10.1073/pnas.1015245107 21127264 PMC3009830

[22] West KA, Castillo SS, Dennis PA. Activation of the PI3K/Akt pathway and chemotherapeutic resistance. Drug Resist Updat. 2002; 5:234–48. 10.1016/S1368-7646(02)00120-6 12531180

[23] Geoerger B, Kerr K, Tang CB, Fung KM, Powell B, Sutton LN, Phillips PC, Janss AJ. Antitumor activity of the rapamycin analog CCI-779 in human primitive neuroectodermal tumor/medulloblastoma models as single agent and in combination chemotherapy. Cancer Res. 2001; 61:1527–32. 11245461

[24] Xu Q, Simpson SE, Scialla TJ, Bagg A, Carroll M. Survival of acute myeloid leukemia cells requires PI3 kinase activation. Blood. 2003; 102:972–80. 10.1182/blood-2002-11-3429 12702506

[25] Shi Y, Frankel A, Radvanyi LG, Penn LZ, Miller RG, Mills GB. Rapamycin enhances apoptosis and increases sensitivity to cisplatin *in vitro*. Cancer Res. 1995; 55:1982–88. 7728769

[26] Rickles RJ, Matsui J, Zhu P, Funahashi Y, Grenier JM, Steiger J, Zhao N, Littlefield BA, Nomoto K, Uenaka T. Identification of Combinatorial Drugs that Synergistically Kill both Eribulin-Sensitive and Eribulin-Insensitive Tumor Cells. Glob J Cancer Ther. 2015; 1:009-17 https://doi.org/10.17352/gjct.000004.

[27] Mundt F, Rajput S, Li S, Ruggles KV, Mooradian AD, Mertins P, Gillette MA, Krug K, Guo Z, Hoog J, Erdmann-Gilmore P, Primeau T, Huang S, et al. Mass Spectrometry-Based Proteomics Reveals Potential Roles of NEK9 and MAP2K4 in Resistance to PI3K Inhibition in Triple-Negative Breast Cancers. Cancer Res. 2018; 78:2732–46. 10.1158/0008-5472.CAN-17-1990 29472518 PMC5955814

[28] Ricardo S, Vieira AF, Gerhard R, Leitão D, Pinto R, Cameselle-Teijeiro JF, Milanezi F, Schmitt F, Paredes J. Breast cancer stem cell markers CD44, CD24 and ALDH1: expression distribution within intrinsic molecular subtype. J Clin Pathol. 2011; 64:937–46. 10.1136/jcp.2011.090456 21680574

[29] Han J, Fujisawa T, Husain SR, Puri RK. Identification and characterization of cancer stem cells in human head and neck squamous cell carcinoma. BMC Cancer. 2014; 14:173. https://doi.org/10.1186/1471-2407-14-173 24612587 10.1186/1471-2407-14-173 PMC4008349

[30] Baumann P, Cremers N, Kroese F, Orend G, Chiquet-Ehrismann R, Uede T, Yagita H, Sleeman JP. CD24 expression causes the acquisition of multiple cellular properties associated with tumor growth and metastasis. Cancer Res. 2005; 65:10783–93. 10.1158/0008-5472.CAN-05-0619 16322224

[31] Dent R, Trudeau M, Pritchard KI, Hanna WM, Kahn HK, Sawka CA, Lickley LA, Rawlinson E, Sun P, Narod SA. Triple-negative breast cancer: clinical features and patterns of recurrence. Clin Cancer Res. 2007; 13:4429–34. 10.1158/1078-0432.CCR-06-3045 17671126

[32] Liedtke C, Mazouni C, Hess KR, André F, Tordai A, Mejia JA, Symmans WF, Gonzalez-Angulo AM, Hennessy B, Green M, Cristofanilli M, Hortobagyi GN, Pusztai L. Response to neoadjuvant therapy and long-term survival in patients with triple-negative breast cancer. J Clin Oncol. 2008; 26:1275–81. 10.1200/JCO.2007.14.4147 18250347

[33] Network TC, and Cancer Genome Atlas Network. Comprehensive molecular portraits of human breast tumours. Nature. 2012; 490:61–70. 10.1038/nature11412 23000897 PMC3465532

[34] Dourdin N, Schade B, Lesurf R, Hallett M, Munn RJ, Cardiff RD, Muller WJ. Phosphatase and tensin homologue deleted on chromosome 10 deficiency accelerates tumor induction in a mouse model of ErbB-2 mammary tumorigenesis. Cancer Res. 2008; 68:2122–31. 10.1158/0008-5472.CAN-07-5727 18381417 PMC2752841

[35] Saal LH, Gruvberger-Saal SK, Persson C, Lövgren K, Jumppanen M, Staaf J, Jönsson G, Pires MM, Maurer M, Holm K, Koujak S, Subramaniyam S, Vallon-Christersson J, et al. Recurrent gross mutations of the PTEN tumor suppressor gene in breast cancers with deficient DSB repair. Nat Genet. 2008; 40:102–07. 10.1038/ng.2007.39 18066063 PMC3018354

[36] Kim C, Gao R, Sei E, Brandt R, Hartman J, Hatschek T, Crosetto N, Foukakis T, Navin NE. Chemoresistance Evolution in Triple-Negative Breast Cancer Delineated by Single-Cell Sequencing. Cell. 2018; 173:879–893.e13. 10.1016/j.cell.2018.03.041 29681456 PMC6132060

[37] Mondesire WH, Jian W, Zhang H, Ensor J, Hung MC, Mills GB, Meric-Bernstam F. Targeting mammalian target of rapamycin synergistically enhances chemotherapy-induced cytotoxicity in breast cancer cells. Clin Cancer Res. 2004; 10:7031–42. 10.1158/1078-0432.CCR-04-0361 15501983

[38] Wong SW, Tiong KH, Kong WY, Yue YC, Chua CH, Lim JY, Lee CY, Quah SI, Fow C, Chung C, So I, Tan BS, Choo HL, et al. Rapamycin synergizes cisplatin sensitivity in basal-like breast cancer cells through up-regulation of p73. Breast Cancer Res Treat. 2011; 128:301–13. 10.1007/s10549-010-1055-0 20686837

[39] Hirai H, Sootome H, Nakatsuru Y, Miyama K, Taguchi S, Tsujioka K, Ueno Y, Hatch H, Majumder PK, Pan BS, Kotani H. MK-2206, an allosteric Akt inhibitor, enhances antitumor efficacy by standard chemotherapeutic agents or molecular targeted drugs *in vitro* and *in vivo*. Mol Cancer Ther. 2010; 9:1956–67. 10.1158/1535-7163.MCT-09-1012 20571069

[40] Morgillo F, Della Corte CM, Diana A, Mauro CD, Ciaramella V, Barra G, Belli V, Franzese E, Bianco R, Maiello E, de Vita F, Ciardiello F, Orditura M. Phosphatidylinositol 3-kinase (PI3Kα)/AKT axis blockade with taselisib or ipatasertib enhances the efficacy of anti-microtubule drugs in human breast cancer cells. Oncotarget. 2017; 8:76479–91. 10.18632/oncotarget.20385 29100327 PMC5652721

[41] Asano M, Matsui J, Towle MJ, Wu J, McGonigle S, DE Boisferon MH, Uenaka T, Nomoto K, Littlefield BA. Broad-spectrum Preclinical Antitumor Activity of Eribulin (Halaven®): Combination with Anticancer Agents of Differing Mechanisms. Anticancer Res. 2018; 38:3375–85. 10.21873/anticanres.12604 29848686

[42] Serra V, Gris-Oliver A, Saura C, Oliveira M, Piris A, Ibrahim Y, Prudkin L, Pérez-García JM, Baselga J, Cortés J. Abstract P5-08-06: PI3K blockade enhances the antitumor activity of eribulin in PIK3CA-mutant eribulin-resistant tumor xenografts. Cancer Res. 2013; 73:P5-08-06 https://doi.org/10.1158/0008-5472.SABCS13-P5-08-06.

[43] Gonzalez-Angulo AM, Green MC, Murray JL, Palla SL, Koenig KH, Brewster AM, Valero V, Ibrahim NK, Moulder SL, Litton JK, Crawford DJ, Flores PR, Dryden MJ, et al. Open label, randomized clinical trial of standard neoadjuvant chemotherapy with paclitaxel followed by FEC (T-FEC) versus the combination of paclitaxel and RAD001 followed by FEC (TR-FEC) in women with triple receptor-negative breast cancer (TNBC). J Clin Oncol. 2011; 29:1016 https://doi.org/10.1200/jco.2011.29.15_suppl.1016. 24669015 10.1093/annonc/mdu124 PMC4037860

[44] Tripathy D, Chien AJ, Hylton N, Buxton MB, Ewing CA, Wallace AM, Forero A, Kaplan HG, Nanda R, Albain KS, Moulder SL, Haley BB, DeMicheleet A, et al Adaptively randomized trial of neoadjuvant chemotherapy with or without the Akt inhibitor MK-2206: Graduation results from the I-SPY 2 Trial. J Clin Oncol. 2015; 33:524 10.1200/jco.2015.33.15_suppl.52425584001

[45] Kim SB, Dent R, Im SA, Espié M, Blau S, Tan AR, Isakoff SJ, Oliveira M, Saura C, Wongchenko MJ, Kapp AV, Chan WY, Singel SM, et al, and LOTUS investigators. Ipatasertib plus paclitaxel versus placebo plus paclitaxel as first-line therapy for metastatic triple-negative breast cancer (LOTUS): a multicentre, randomised, double-blind, placebo-controlled, phase 2 trial. Lancet Oncol. 2017; 18:1360–72. 10.1016/S1470-2045(17)30450-3 28800861 PMC5626630

[46] Ailles L, Prince M. Cancer stem cells in head and neck squamous cell carcinoma. Methods Mol Biol. 2009; 568:175–93. 10.1007/978-1-59745-280-9_11 19582427

[47] Dean M, Fojo T, Bates S. Tumour stem cells and drug resistance. Nat Rev Cancer. 2005; 5:275–84. 10.1038/nrc1590 15803154

[48] Calcagno AM, Salcido CD, Gillet JP, Wu CP, Fostel JM, Mumau MD, Gottesman MM, Varticovski L, Ambudkar SV. Prolonged drug selection of breast cancer cells and enrichment of cancer stem cell characteristics. J Natl Cancer Inst. 2010; 102:1637–52. 10.1093/jnci/djq361 20935265 PMC2970576

[49] Hu Y, Guo R, Wei J, Zhou Y, Ji W, Liu J, Zhi X, Zhang J. Effects of PI3K inhibitor NVP-BKM120 on overcoming drug resistance and eliminating cancer stem cells in human breast cancer cells. Cell Death Dis. 2015; 6:e2020. https://doi.org/10.1038/cddis.2015.363 26673665 10.1038/cddis.2015.363 PMC4720896

[50] Zhou J, Wulfkuhle J, Zhang H, Gu P, Yang Y, Deng J, Margolick JB, Liotta LA, Petricoin E 3rd, Zhang Y. Activation of the PTEN/mTOR/STAT3 pathway in breast cancer stem-like cells is required for viability and maintenance. Proc Natl Acad Sci USA. 2007; 104:16158–63. 10.1073/pnas.0702596104 17911267 PMC2042178

[51] Airiau K, Mahon FX, Josselin M, Jeanneteau M, Belloc F. PI3K/mTOR pathway inhibitors sensitize chronic myeloid leukemia stem cells to nilotinib and restore the response of progenitors to nilotinib in the presence of stem cell factor. Cell Death Dis. 2013; 4:e827. 10.1038/cddis.2013.309 24091670 PMC3824646

[52] Wang XQ, Ongkeko WM, Chen L, Yang ZF, Lu P, Chen KK, Lopez JP, Poon RT, Fan ST. Octamer 4 (Oct4) mediates chemotherapeutic drug resistance in liver cancer cells through a potential Oct4-AKT-ATP-binding cassette G2 pathway. Hepatology. 2010; 52:528–39. 10.1002/hep.23692 20683952

[53] Zhang X, Zhang S, Liu Y, Liu J, Ma Y, Zhu Y, Zhang J. Effects of the combination of RAD001 and docetaxel on breast cancer stem cells. Eur J Cancer. 2012; 48:1581–92. 10.1016/j.ejca.2012.02.053 22420943

[54] Ma CX, Cai S, Li S, Ryan CE, Guo Z, Schaiff WT, Lin L, Hoog J, Goiffon RJ, Prat A, Aft RL, Ellis MJ, Piwnica-Worms H. Targeting Chk1 in p53-deficient triple-negative breast cancer is therapeutically beneficial in human-in-mouse tumor models. J Clin Invest. 2012; 122:1541–52. 10.1172/JCI58765 22446188 PMC3314455

