# Supplementary material for: PI3K inhibition enhances the anti-tumor effect of eribulin in triple negative breast cancer
Source: Oncotarget. 2019 Jun 4;10(38):3667–80. (PMC6557212)
Supplement: Supplementary file 1 [file oncotarget-10-3667-s001.pdf]

PI3K inhibition enhances the Anti-tumor effect of eribulin in triple negative breast cancer

SUPPLEMENTARY MATERIALS

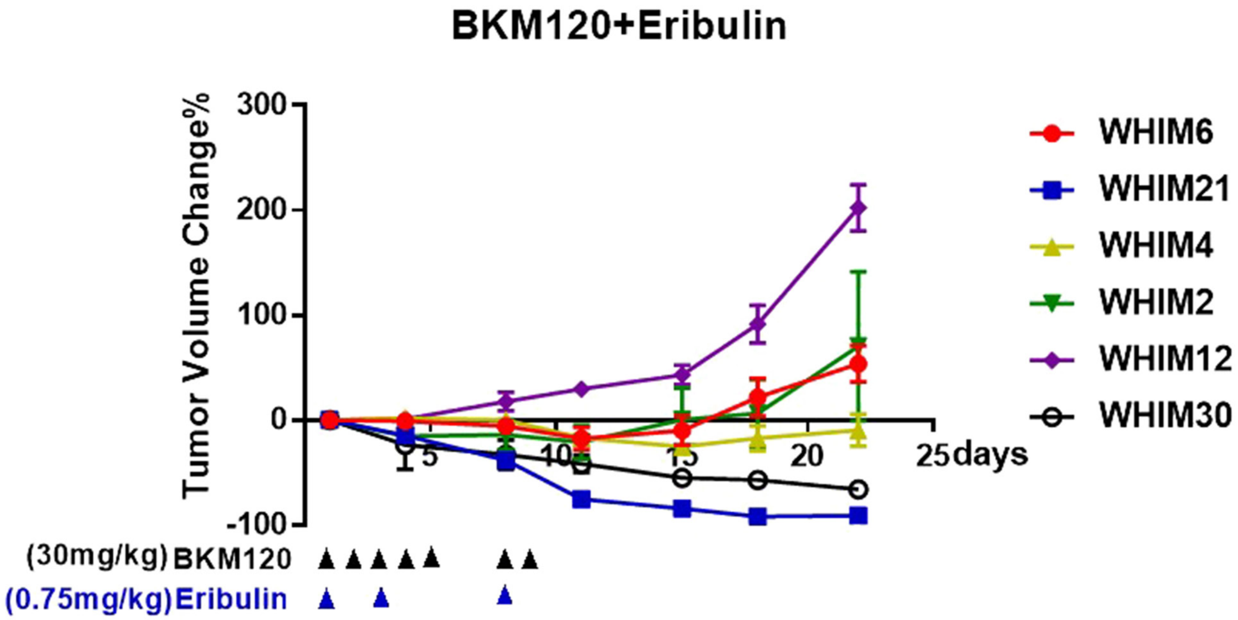

Supplementary Figure 1: Screening experiment assessing the efficacy of eribulin in combination with BKM120 in 6 TNBC PDXs. Tumor volume changes with time compared to baseline following treatment with eribulin (0.75 mg/kg, IP, days 1, 3 and 8) in combination with BKM120 (30 mg/kg, PO, days 1-5 then days 7-8) for each PDX models were shown (n=1-3 per group).

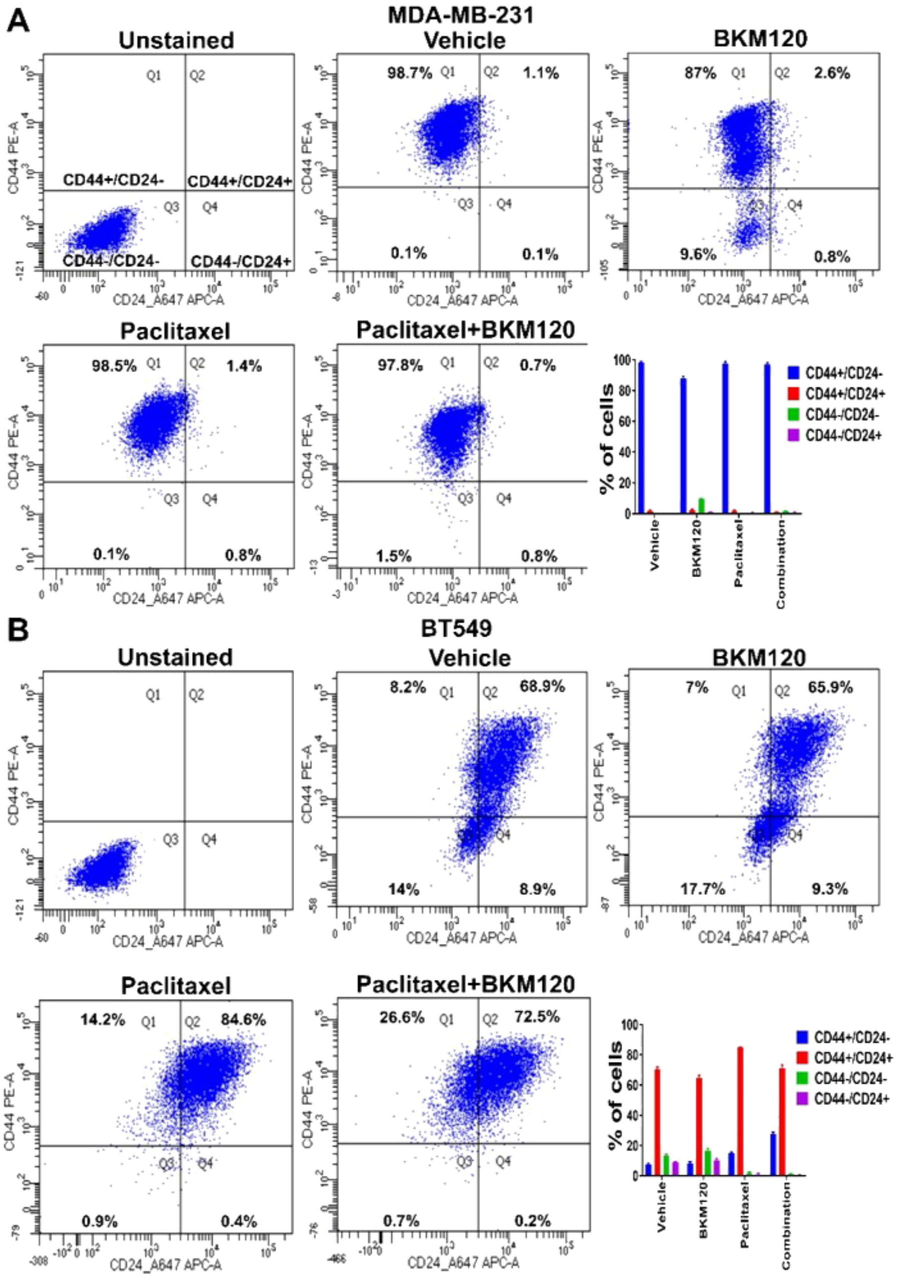

Supplementary Figure 2: Paclitaxel does not inhibit the stem cell population in TNBC lines *in vitro*. Representative FACS analysis of stem cell population in **(A)** MDA-MB-231 and **(B)** BT549 cells after treatment with either vehicle, or paclitaxel and BKM120, either alone or in combination for 48 hours by FACS. Gates were adjusted using unstained and isotype secondary controls. **(A)** and **(B)** show the quantification of cellular subsets by CD44 and CD24 staining for MDA-MB-231 and BT549, respectively, from three independent experiments.
